# Development of an in vitro model to study clot lysis activity of thrombolytic drugs

**DOI:** 10.1186/1477-9560-4-14

**Published:** 2006-09-12

**Authors:** Sweta Prasad, Rajpal S Kashyap, Jayant Y Deopujari, Hemant J Purohit, Girdhar M Taori, Hatim F Daginawala

**Affiliations:** 1 Biochemistry Research Laboratory, Central India Institute of Medical Sciences, 88/2 Bajaj Nagar, Nagpur-440010, India; 2 Environmental Genomics Unit, NEERI, Nehru Marg, Nagpur-440020, India

## Abstract

**Background:**

Thrombolytic drugs are widely used for the management of cerebral venous sinus thrombosis patients. Several in vitro models have been developed to study clot lytic activity of thrombolytic drugs, but all of these have certain limitations. There is need of an appropriate model to check the clot lytic efficacy of thrombolytic drugs. In the present study, an attempt has been made to design and develop a new model system to study clot lysis in a simplified and easy way using a thrombolytic drug, streptokinase.

**Methods:**

Whole blood from healthy individuals (*n *= 20) was allowed to form clots in a pre-weighed sterile microcentrifuge tubes; serum was removed and clot was weighed. After lysis by streptokinase fluid was removed and remnants of clot were again weighed along with the tube. Percentage of Clot lysis was calculated on the basis of the weight difference of microcentrifuge tubes obtained before and after clot lysis.

**Results:**

There was a significant percentage of clot lysis observed when streptokinase was used. On the other hand with water (negative control), minimal (2.5%) clot lysis was observed. There was a significant difference between clot lysis done by streptokinase and water.

**Conclusion:**

Our study could be a rapid and effective methodology to study clot-lytic effect of newly developed drugs as well as known drugs.

## Background

Cerebral venous sinus thrombosis (CVST) is a common disorder that is often accompanied by significant morbidity and mortality [1,2]. In anticoagulation therapy the intravenous heparin [2,3] is the first line of treatment for CVST, because of its efficacy, safety and feasibility. However, thrombolytic therapy, with its ability to produce rapid clot lysis, has long been considered an attractive alternative [4]. Thrombolytic drugs like tissue plasminogen activator (t-PA), urokinase, streptokinase etc. play a crucial role in the management of patients with CVST. In India though streptokinase and urokinase are widely used due to its lower cost, as compared to other thrombolytic drugs, these are dangerous [5-9] because they might cause serious bleeding complications along with reocclusion and reinfarction.

Various methods were developed to measure the clot lysis activity of thrombolytic drugs. The best way to study thrombolytic drugs is through in vitro clot lysis model [10-19]. Earlier *Basta et al *[10] worked on artificial clots and used ultrasound methods to measure the thrombolytic activity of streptokinase. Several other models have been reported which either uses complicated mathematical [11] or computing [12] skills, but all these methods are very costly and not affordable in developing countries. The above-mentioned problems demand a need of simple and cost effective clot lytic model for measurement of clot lysis activity of thrombolytic drugs. In the present study an attempt has been made to develop an in-vitro clot lytic model using a known thrombolytic drug, streptokinase.

## Materials and methods

### Specimen

Venous blood was drawn from healthy human volunteers (*n *= 20) without a history of oral contraceptive or anticoagulant therapy (using a protocol approved by our Institutional Ethics Committee). 500 μl of blood was transferred to each of the previously weighed microcentrifuge tubes to form clots.

### Streptokinase

To the commercially available lyophilized streptokinase vial (15, 00,000 I.U.) 5 ml phosphate buffered saline (PBS) was added and mixed properly. This suspension was used as a stock from which appropriate dilutions were made to observe the thrombolytic activity using the in vitro model developed in our laboratory.

### Study design

Venous blood drawn from healthy volunteers (*n *= 20) was transferred in different pre weighed sterile microcentrifuge tube (500 μl/tube) and incubated at 37°C for 45 minutes. After clot formation, serum was completely removed (aspirated out without disturbing the clot formed) and each tube having clot was again weighed to determine the clot weight (clot weight = weight of clot containing tube – weight of tube alone). Each microcentrifuge tube containing clot was properly labeled and 100 μl of streptokinase along with various dilutions in sterile distilled water (undiluted, 3:4, 1:2 and 1:3) was added to the tubes. Water was also added to one of the tubes containing clot and this serves as a negative thrombolytic control. All the tubes were then incubated at 37°C for 90 minutes and observed for clot lysis. After incubation, fluid obtained was removed and tubes were again weighed to observe the difference in weight after clot disruption. Difference obtained in weight taken before and after clot lysis was expressed as percentage of clot lysis. The test was repeated ten times with all the four dilutions of the thrombolytic drugs (Streptokinase) in blood samples of twenty different healthy volunteers.

### Statistical analysis

The mean clot lysis percent of streptokinase with different concentrations was compared with water using the repeated measures ANOVA with Tukey post test. Data are expressed as mean ± standard deviation. A p value ≤ 0.05 was considered to be statistically significant.

## Results

A clear, visual representation of clot lysis is shown in figure no. 1. When 100 μl water was added to the control clot negligible clot lysis was observed. Whereas, tubes to which different dilutions of streptokinase was added, significant clot lysis could be visually seen. Percent clot lysis obtained after treating clots with streptokinase and control group is shown in figure 2. Maximum clot lysis was observed when undiluted streptokinase (100 μl) was added to the clots. With water 2.55% weight difference was seen. Though undiluted streptokinase (30,000 I.U.) showed maximum clot lysis (p = 0.004) but another three dilutions (22,500 I.U., 15,000 I.U., 7,500 I.U.) of streptokinase also showed approximately same percent of clot lysis (p < 0.05). The mean clot lysis % of streptokinase with all different concentration was found to differ significantly when compared with water (i.e, p < 0.05 for all the concentrations of streptokinase).

**Figure 1:**
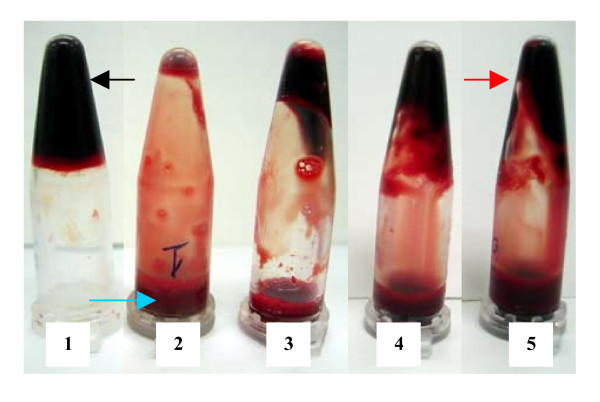
**Clot-lysis of blood samples of normal subjects (positive and negative control)**. Tube no. 1 is a control clot (negative control) to which water was added. No clot lysis was observed in tube no.1; a black arrow indicates the intact clot. Tube no. 2–5 (positive control) was lysed by four different concentrations of streptokinase with decreasing order. After dissolution of the clots, tubes were inverted and fluid (blue arrow) along with the remnants of clots (red arrow) could be clearly seen

**Figure 2:**
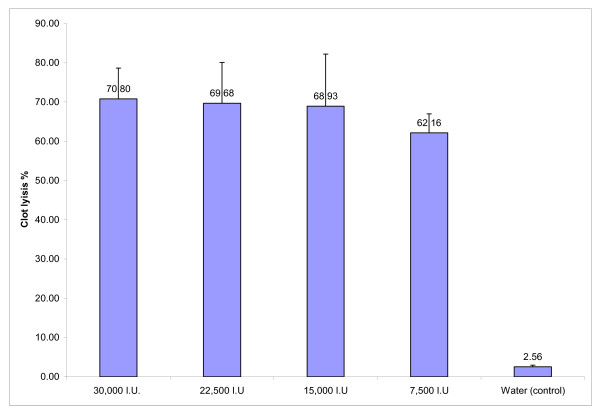
**Clot lysis of blood samples of normal subjects by different concentrations of Streptokinase**. Effect of streptokinase on the dissolution of the clots prepared with blood of normal subjects. The clots were treated by four different dilutions of streptokinase i.e., 30,000 I.U, 22,500 I.U., 15,000 I.U., and 7,500 I.U.) and clot lysis % was 70.80, 69.68, 68.93 and 62.16 respectively. Clot lysis % by each dilution of streptokinase differs significantly with water (control) (p < 0.05). The percentage of clot lysis by different concentration of streptokinase ranges from 62–71%

## Discussion

This study shows in vitro dissolution of clots by four different dilutions of streptokinase, assayed by an in vitro clot lysis model designed and developed in our laboratory. Most of the in vitro methods that were conventionally or currently applied to study thrombolysis have certain limitations. Some involve complex computation and mathematical skills that too give only theoretical prediction of the outcome and most are expensive to be performed in a laboratory. In context with the current scenario we thought of developing a clot lysis model that would be easy to perform & that should be cost effective too.

Keeping this idea in the prime focus, weight of the clot before lysis and after lysis was considered as appropriate determinant of calculating clot lysis percentage. Blood collected by the standard protocol of venipuncture was allowed to form clots naturally and the weight difference obtained before and after lysis was noted and clot lysis percentage was calculated. In other methods, there are different parameters to analyze the extent of clot lysis. For example, radiolabelling of factors that are actively involved in clot lysis mechanism [13], MRI [15], ultrasound frequency [10], turbidity determination using microtiter plate reader (euglobulin lysis test) [16,17], study of fibrinolytic activity by circulating fibrinolytic enzymes and monitoring the effect by calculating shear rate [18,19]. All these methods are sophisticated whereas, our model is simple and easy to perform and one can even visually observe the lysis of clots (as shown in figure 1).

## Conclusion

To check the efficacy of thrombolytic drugs or herbs one can compare the data with positive and negative control. In our study we took a known thrombolytic drug; streptokinase as a positive control and water as a negative control. Test drug can also be analyzed in the same way. Thus, our study could be an instant and effective methodology to study clot lytic effect of newly developed drugs as well as known drugs.

## Declaration of competing interests

The author(s) declare that they have no competing interests.

## Authors' contributions

SP carried out the study design, data collection, literature search, statistical analysis and manuscript preparation; RSK, JYD, HJP participated in the preparation of the manuscript, data interpretation, and study design; GMT provided assistance in preparation of the manuscript, data interpretation, study design, and funds collection; and HFD supervised the study design, data interpretation, manuscript preparation, and literature search. All authors have read and approved the final version of the manuscript.
